# Special Issue: Advanced Science and Technology of Polymer Matrix Nanomaterials

**DOI:** 10.3390/ma15144735

**Published:** 2022-07-06

**Authors:** Liguo Xu, Jintang Zhou, Zibao Jiao, Peijiang Liu

**Affiliations:** 1 College of Light Chemical Industry and Materials Engineering, Shunde Polytechnic, Foshan 528333, China; 21099@sdpt.edu.cn; 2 College of Materials Science and Technology, Nanjing University of Aeronautics and Astronautics, Nanjing 211100, China; 3 Reliability Research and Analysis Centre, China Electronic Product Reliability and Environmental Testing Research Institute, Guangzhou 510610, China

Nanotechnology has witnessed an incredible resonance and a substantial number of new applications in various areas during the past three decades [1]. The resulting basic paradigm shifts have opened up new possibilities towards materials science, and have caused dramatic developments. Basically, nanotechnology necessarily relies on the presence or supply of novel nanomaterials that form the prerequisite for any ongoing progress in this interdisciplinary area of technology and science [2]. Among other nanomaterials, in the quest for eliminating the inherent shortcomings of pristine polymers, polymer matrix nanomaterials are fabricated through the introduction of nanomaterials with uniform distribution in pure polymer matrices [3,4].

Polymer matrix nanomaterial is, thereby, an active coupling of nanomaterials (other fillers may also be present) and polymers, where at least one phase is preserved in the nanosized regime (within 100 nm) in the resultant materials. As the existence of nanomaterials in the polymer matrix features particular properties that are characteristic of this kind of material, and that are correlative with surface and quantum effects, it may intrinsically develop a fresh set of properties hinging on the nanomaterials utilized. Additionally, nanomaterials supply a significant number of interfacial areas within the matrix and accordingly at sufficiently low concentrations improve the material properties, which implies lowering the product gross weight further. Therefore, the union of nanotechnology and nanoscience with polymer technology and science has accelerated multifaceted application-oriented uses for polymer matrix nanomaterials. Numerous studies have revealed that the conductivity, mechanical, magnetic, optical, dielectric, electronic and biological characteristics of several inorganic nanomaterials dramatically change as their sizes reduce from the macroscale to the micro and nano scale. In the field of polymer matrix nanomaterials, researchers and experts have been focusing on the reinforced characteristics (mechanical strength, impact resistance, conductivity, biodegradability) and many diverse functionalities (self-healing, anti-fouling, electro-optical properties, flame resistance, controlled substance release, energy absorption applications and others) that afford them with certain properties, performance, or applications of considerable industrial interest. Therefore, polymer matrix nanomaterials have engraved an inimitable role in the niche of advanced materials and technologies. A genius multidisciplinary cooperation of material science with physics, biology, chemistry, nanotechnology, engineering, and medical science is inevitable for the actual exploration of such a new class of materials. In this regard, the development of modulated polymer systems will enable us to tackle technical and scientific challenges at the same time as satisfying the globally increasing demand.

The current Special Issue entitled “Advanced Science and Technology of Polymer Matrix Nanomaterials” is engaged in uniting researchers and scientists working at research institutes, laboratories, universities and industries to discuss cutting-edge developments and research on processing new polymer matrix nanomaterials in which nanoscale particle materials, including graphene, single-walled and multiwalled carbon nanotubes, inorganic layered clay, metal and metal oxide nanoparticles, MXene and others, have been introduced [5,6,7,8]. The subjects of this issue aim to uncover the potential improvements in the synthesis, properties and performance of polymer matrix nanomaterials regarding new preparation techniques, sensing, electromagnetic interference shielding, self-healing, microwave absorption, switching, structural modulation, mechanical reinforcement, drug delivery and other biomedical applications etc. [9,10,11,12,13,14,15,16].

As Guest Editors, it is our honor to invite contributions in the form of original research articles or reviews about this subject.
